# Efficacy and prognostic factors of repeated hepatectomy for postoperative intrahepatic recurrence of hepatocellular carcinoma undergoing initial hepatectomy

**DOI:** 10.3389/fmed.2023.1127122

**Published:** 2023-05-11

**Authors:** Feng Cen, Xu Sun, Zhiping Pan, Qiang Yan

**Affiliations:** ^1^Department of Hepatobiliary and Pancreatic Surgery, Huzhou Central Hospital, Huzhou, China; ^2^The Affiliated Huzhou Hospital, Zhejiang University School of Medicine, Huzhou, China; ^3^Huzhou Key Laboratory of Intelligent and Digital Precision Surgery, Huzhou Central Hospital, Huzhou, China

**Keywords:** hepatectomy, hepatocellular carcinoma, intrahepatic recurrence, independent prognosis, recurrent hepatocellular carcinoma, radiofrequency ablation

## Abstract

**Background:**

Postoperative recurrence of hepatocellular carcinoma (HCC) is associated with low survival rates. While HCC treatment options have expanded substantially, they are accompanied by several challenges. This study assessed the outcomes of repeated hepatectomy (RH) for postoperative intrahepatic recurrence of HCC among patients undergoing initial hepatectomy (IH) as well as independent risk factors for HCC recurrence among patients undergoing repeated hepatectomy (RH).

**Methods:**

Clinical data from 84 patients undergoing both IH and RH and 66 recurrent HCC patients who had received radiofrequency ablation (RFA) from July 2011 to September 2017 were retrospectively reviewed. The following groups were compared: (1) RH Group A (*n* = 84), (2) IH Group (*n* = 84, same individuals as RH Group A), (3) RH Group B (*n* = 45/84 from RH Group A), and (4) RFA Group (*n* = 66). The clinical pathology and operative characteristics of the patients in RH Group A were compared to those in the IH Group. Meanwhile, the clinical pathology and pre- and post-treatment features of the patients in RH Group B were compared to those in the RFA Group. The tumor-free survival time was compared between patients in RH Group A and the IH Group as well as between patients in RH Group B and the RFA Group. The independent risk factors for the 1-year postoperative tumor-free survival of RH Group A patients were investigated using univariate and multivariate analysis.

**Results:**

Measures of clinical pathology, including AFP, Child-Pugh score, HBV-DNA, tumor number, liver cirrhosis, tumor differentiation, surgical approach, and TNM stage differed significantly between patients in RH Group A and the IH Group (all *P* < 0.05), with the exception of tumor number and tumor size (both *P* > 0.05). No significant differences were found in these measures between the patients in RH Group B and the RFA Group (all *P* > 0.05). While patients in the RH Group A had a longer operation time than those in the IH Group (4.35 ± 1.25 h vs. 3.55 ± 0.92 h, *P* < 0.001), the level of intraoperative bleeding was similar (400.00 ± 199.25 ml vs. 359.40 ± 213.37 ml, *P* = 0.204). RH Group B patients had a longer hospitalization time than those in the RFA Group (6.5 ± 0.8 d vs. 5.5 ± 1.1 d, *P* < 0.001), however, the difference in hospitalization costs was not statistically significant (29,009 ± 3,806 CNY vs. 29,944 ± 3,752 CNY, *P* = 0.202). Five-day post-operative serum biomarker levels, including direct bilirubin (DB) and albumin (ALB), were significantly higher in RH Group B than in the RFA Group (all *P* < 0.05), with the exception of ALT, AST, and total bilirubin (TB) (all *P* > 0.05). Patients in RH Group A had a lower tumor-free survival time than those in the IH Group (median: 12 vs. 22 months, *P* < 0.001), and patients in the RH Group B had a significantly higher tumor-free survival time than those in the RFA group (median: 15 months vs. 8 months, *P* < 0.001). Age ≥50 y, Child-Pugh class A, and negative HBV-DNA were independent risk factors that positively impacted the 1-year postoperative tumor-free survival rate of postoperative intrahepatic recurrent HCC patients undergoing RH (*P* < 0.001, respectively).

**Conclusion:**

Due to the potential of harm related to relapse of recurrent HCC for cancer patients, RH is a superior option. RH could offer better outcomes for recurrent HCC patients undergoing IH. Compared with lesion pathology, the better target organ of the liver will be key to ameliorating tumor-free survival for recurrent HCC patients undergoing RH.

## 1. Introduction

Hepatocellular carcinoma (HCC), which accounts for more than 90% of primary liver cancers, is the fourth leading cause of cancer-related mortality worldwide and a leading cause of cirrhosis-related death (1). HCC prognosis is poor, with the global mortality rates approaching the incidence rates (2). In 2018, the estimated global incidence rate of liver cancer per 100,000 person-years was 9.3 while the corresponding mortality rate was 8.5 (3). In recent years, there have been groundbreaking advancements in HCC treatment, particularly among advanced-stage patients. Radical resection, the preferred treatment for HCC, has a 5-year overall survival (OS) rate of about 50% (4–6). However, the 5-year recurrence rate is 60–100% after hepatectomy (4, 7, 8) which is critical to consider for the development of an optimal treatment plan. Cirrhosis patients with a significant degree of liver dysfunction are usually unsuitable for RH due to the lack of adequate hepatic functional reserve caused by IH. Thus, RH should only be performed according to specified indications. The current study sought to assess the value of RH for HCC patients with postoperative intrahepatic recurrence who underwent IH and to investigate the independent risk factors of postoperative tumor-free survival among individuals with recurrent HCC who received RH.

## 2. Materials and methods

### 2.1. Patients and groups

This was a retrospective cohort study that spanned a 6-year period from July 2011 to September 2017. The study included the clinical data from 84 recurrent HCC patients who underwent curative repeated hepatectomy (RH Group A) and the same individuals with initial HCC who received an initial hepatectomy (IH Group) in the Department of Hepatobiliary Surgery at Huzhou Central Hospital/The Affiliated Huzhou Hospital, Zhejiang University School of Medicine. The IH Group included 61 patients with single lesions and 23 patients with multiple lesions while the RH Group A included 39 patients with single lesions and 45 patients with multiple lesions. The IH Group and RH Group A had 12 and five patients, respectively, with a single lesion size of >5 cm along with multiple lesions. A total of 45 patients were the constituent part of RH Group A (45/84; RH Group B) with ≤ 3 tumors that were ≤ 3 cm in diameter. An additional 66 patients had a postoperative recurrence of HCC and were treated with RFA (RFA Group). RFA was used on patients with ≤ 3 tumors that were ≤ 3 cm in diameter to improve the tumor-free survival time until recurrence.

All enrolled patients had received a medical imaging examination (i.e., CT, MRI, PET-CT) to exclude extrahepatic lesions or vascular invasion such as portal vein tumor thrombosis (PVTT) during IH, RH, or RFA treatment. Individuals with advanced liver disease who were treated with a systemic treatment, such as immunotherapy (immune checkpoint inhibitors, ICIs), tyrosine kinase inhibitors (TKIs), and anti-VEGF therapies, were excluded. Staging of recurrent HCC was dependent on performance status (PS), degree of liver dysfunction, and traditional TNM staging. Cancer stages were classified according to the American Joint Committee on Cancer/Union for International Cancer Control (AJCC/UICC) TNM staging system (9). The study was reviewed and approved by the Medical Ethics Committee of Huzhou Central Hospital and the application for informed patient consent was exempted.

Clinical, pathological, operative, and pre-/post-treatment characteristics and tumor-free survival time were assessed and compared between patients in the RH Group A and the IH Group, and between patients in the RH Group B and RFA Group. The independent risk factors of 1-year postoperative tumor-free survival of patients in the RH Group A were investigated using univariate and multivariate analysis.

### 2.2. Diagnosis of recurrent HCC

All postoperative individuals received regular clinical follow-up visits once a month for the first 6 months and once every 3 months for the second 6 months. Follow-up by outpatient examination was considered until October 1, 2017, with first recurrence defined as the endpoint. The Liver Reporting and Data System (LIRADS) allows for the standardized classification of liver lesions in cirrhosis patients. Follow-up visits included ultrasonography (US), CT, MRI, PET-CT, and measurements of serum biomarkers such as AFP, HBV-DNA, and the liver function index. Recurrence was diagnosed by the presence of new lesions on imaging with a typical appearance of HCC or a rise in serum AFP of ≥400 ng/ml determined by radioimmunoassay, excluding pregnancy and active liver disease. Multiphase CT or MRI with contrast was performed for definitive diagnosis of intrahepatic lesions through arterial phase enhancement and washout on the portal venous phase as well as multiphase chest CT and enhanced abdominal CT or MRI with contrast. All patients were required to follow up with their doctor if they experienced any discomfort.

### 2.3. Selection criteria for recurrent HCC patients with RH and RFA

Liver function was characterized as compensated or decompensated depending on the presence of jaundice, ascites, and hepatic encephalopathy. Hepatic functional reserve was assessed for recurrent HCC patients without decompensated cirrhosis using the Child-Pugh (CP) scoring system and the Indocyanine Green (ICG) excretive test. Advanced HCC associated with extrahepatic lesions or vascular invasion (i.e., PVTT) was considered a contraindication for resection, thus, considering the benefit to advanced HCC patients, systemic therapy should be performed preferentially. Selected patients with tumors located in a single lobe and an adequate hepatic functional reserve with a Child-Pugh A/B score and an Indocyanine Green Rate at 15 min (ICG-R15) of < 10% were provided RH treatment. According to the liver functional reserve and tumor location, a hemi- or sectional-hepatectomy was conducted for centrally located lesions, and wedge resection was conducted for peripherally located lesions. All liver resections were performed by experienced hepatic surgeons. Tumor size was determined using the maximum diameter for single lesions and the cumulative diameter for multiple lesions. RFA treatment was used for HCC patients with ≤ 3 lesions that were ≤ 3 centimeters in diameter. To ensure successful ablation, the recurrent lesions had to be more centrally located with a sufficient margin of ~10 mm surrounding the tumor. The Cool-tip RF System with power output 0–200 w and a nature frequency 480 kHz produced by American Radionics was used. Power output included 10 w for 30 s or 20–30 w for 150 s for 1 cm lesions, 40 w for 5 min for 2 cm lesions, and 60 w for 8 min for 3 cm lesions. The target temperature for ablation was >65°C for the tumor and 90–99°C for the needle tip, continuing for 5–10 seconds. The total treatment time was 12 min. RFA treatment was performed percutaneously.

### 2.4. Statistical analysis

Data with a normal distribution were analyzed using the Student's *t* test (*t* test) and presented as the mean ± standard deviation (SD). Data with a skewed distribution were analyzed using the non-parametric Mann-Whitney *U*-test and were presented as the *M* (range). Count data were analyzed using the chi-square test (χ^2^ test). The first recurrence was defined as the endpoint, tumor-free survival time was calculated using Bonferroni's correction applied to the Kaplan-Meier method, and the comparison was analyzed using the log-rank test. The risk factors affecting recurrence were analyzed using the Kaplan-Meier method along with Bonferroni's correction for the proportional hazard assumption test, and eligible factors were assessed using the log-rank test during univariate analysis. Multivariate analysis was conducted using the *Cox* proportional hazards model. *P*-values of < 0.05 were considered significant. Statistical analysis was performed using SPSS software version 21.0.

## 3. Results

### 3.1. Comparison between RH for recurrent HCC patients and IH for initial HCC patients

The clinicopathological and operative characteristics were compared between the 84 patients in the IH Group and RH Group A (Table 1). Patients in the IH group were primarily TNM stage I (64 patients) while those in RH Group A were primarily stage I and II (40 and 43 patients, respectively) (*P* < 0.001). There were no obvious differences in the integrity of the tumor capsule and tumor size (n/cm) (*P* = 0.438 and *P* = 0.638/0.074, respectively). However, the remaining characteristics differed significantly between the two groups (all *P* < 0.05). The mean operation time was significantly longer for the patients in RH Group A than those in the IH Group (4.35 ± 1.25 h vs. 3.55 ± 0.92 h, *P* < 0.001). Intraoperative bleeding did not differ significantly between the groups (400.00 ± 199.25 ml vs. 359.40 ± 213.37 ml, *P* = 0.204) (Table 1).

**Table 1:** Comparison between the clinicopathological and operative features of the initial hepatectomy (IH) group and repeated hepatectomy (RH) group A.

**Variables**	**IH group (*n =* 84)**	**RH group A (*n =* 84)**	***χ^2^***	***t***	***P* valve**
**AFP, ng/ml**, ***n***					
< 20	3 (3.6%)	44 (52.4%)	49.659	/	< 0.001
≥20	81 (96.4%)	40 (47.6%)			
**Gender**, ***n***					
Male	66 (78.6%)	66 (78.6%)	/	/	/
Female	18 (21.4%)	18 (21.4%)			
**Child-Pugh**, ***n***					
A	76 (90.5%)	56 (66.7%)	14.141	/	< 0.001
B	8 (9.5%)	28 (33.3%)			
**HBV-DNA**, ***n***					
Negative	36 (42.9%)	62 (73.8%)	16.555	/	< 0.001
Positive	48 (57.1%)	22 (26.2%)			
**Tumor number**, ***n***					
Single	61 (72.6%)	39 (46.4%)	11.958	/	< 0.001
Multiple	23 (27.4%)	45 (53.6%)			
Tumor size^*^, ***n***					
< 5 cm	48 (57.1%)	51 (60.7%)	0.221	/	0.638
≥5 cm	36 (42.9%)	33 (39.3%)			
**Tumor size** ^*^ **, cm**					
mean ± SD	4.926 ± 1.727	4.470 ± 1.555	/	1.798	0.074
**Liver cirrhosis**, ***n***					
Yes	48 (57.1%)	69 (82.1%)	12.416	/	< 0.001
No	36 (42.9%)	15 (17.9%)			
**Tumor capsule**, ***n***					
Complete	40 (47.6%)	35 (41.7%)	0.602	/	0.438
Incomplete	44 (52.4%)	49 (58.3%)			
**Tumor differentiation**, ***n***					
Poor/moderate-poor	42 (50.0%)	58 (69.0%)	6.956	/	0.031
Moderate	34 (40.5%)	23 (27.4%)			
Well/well-moderate	8 (9.5%)	3 (3.6%)			
**Surgical approach**, ***n***					
Regular liver resection	68 (80.9%)	21 (25.0%)	52.782	/	< 0.001
Irregular liver resection	16 (19.1%)	63 (75.0%)			
**AJCC/UICC TNM stage**, ***n***					
I	64 (76.2%)	40 (47.6%)	17.805	/	< 0.001
II	17 (20.2%)	43 (51.2%)			
IIIA	3 (3.6%)	1 (1.2%)			
Operation time, h	3.55 ± 0.92	4.35 ± 1.25	/	1.274	< 0.001
Intraoperative bleeding, ml	359.40 ± 213.37	400.00 ± 199.25	/	4.75	0.204

*n*, Number of patients; ^*^Tumor size was determined by the maximum diameter for single and cumulative diameters for multiple; Cancer stage was classified according to the new AJCC/UICC TNM staging system.

**Figure 1:**
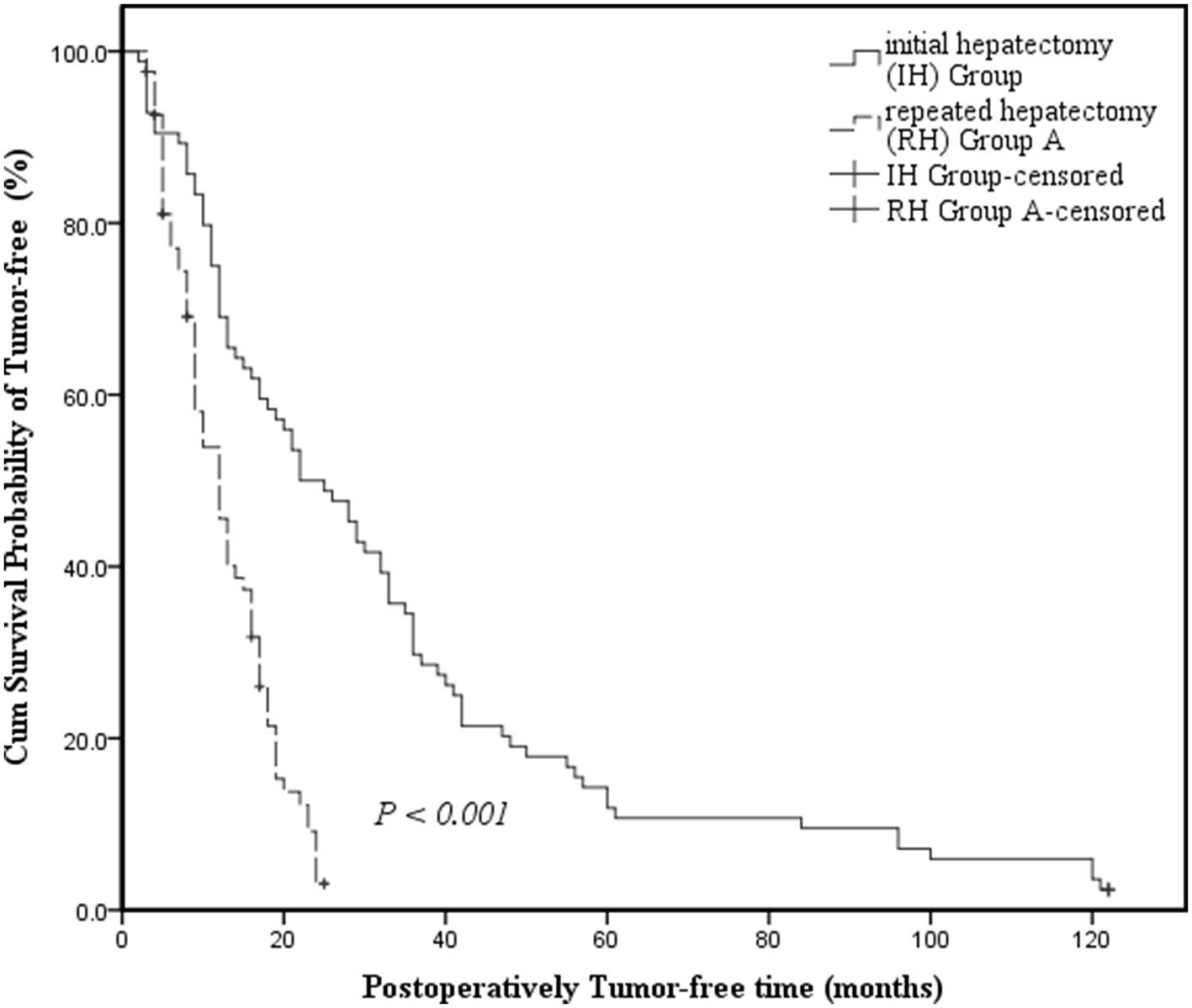
Kaplan-Meier survival curves assessing the cumulative survival incidence of tumor-free survival for patients in the Initial Hepatectomy Group and Repeated Hepatectomy Group A. Cumulative survival incidence was compared between groups using the log-rank test. Kaplan-Meier medians: Initial hepatectomy (IH) Group: 22 months (95% CI: 14.651–29.349); Repeated hepatectomy (RH) Group A: 12 months (95% CI: 9.474–14.526). The tumor-free survival time of the Repeated Hepatectomy Group A is shorter than that of the Initial Hepatectomy Group (log-rank test: χ^2^ = 36.575, *P* < 0.001).

The IH group had a longer median tumor-free survival time than RH Group A and the survival curve revealed a visible difference in the tumor-free survival time between the IH Group and the RH Group A (22 months, 95% CI: 14.651–29.349 vs. 12 months, 95% CI: 9.474–14.526; *P* < 0.001) (Figure 1).

### 3.2. Comparison between RH and RFA for recurrent HCC patients

Pre- and post-treatment clinicopathological characteristics, including postoperative biochemical markers of liver function, were compared between RH Group B and the RFA Group (Tables 2, 3). There were no statistically significant differences in any variables (all *P* > 0.05) (Table 2). While hospitalization time differed significantly between the groups (6.5 ± 0.8 d vs. 5.5 ± 1.1 d, *P* < 0.001), there was no significant difference in hospitalization costs (29,009 ± 3,806 CNY vs. 29,944 ± 3,752 CNY, *P* = 0.202). Serum ALT levels on day 1 and 3, TB on day 1, DB on day 1, 3, and 5, and ALB on day 1 and 5 differed significantly (*P* < 0.001/*P* = 0.003, *P* < 0.001, *P* < 0.001/*P* = 0.037/*P* = 0.018, and *P* = 0.003/*P* = 0.014, respectively). However, there were no obvious differences in serum ALT on day 5, TB on day 3 and 5, AST on day 1, 3, and 5, and ALB on day 3 (all *P* > 0.05) (Table 3).

**Table 2:** Comparison of the clinicopathological and pre-treatment features of repeated hepatectomy (RH) group B and the RFA group.

**Variables**	**RH group B (*n =* 45)**	**RFA group (*n =* 66)**	***χ^2^***	***t***	***P* valve**
Age, y, mean ± SD	53.76 ± 11.53	56.50 ± 10.37	/	−1.308	0.194
**Gender**, ***n***			0.027	/	0.869
Male	30 (66.7%)	43 (65.2%)			
Female	15 (33.3%)	23 (34.8%)			
**AFP, ng/ml**			2.266	/	0.132
< 20	27 (60.0%)	30 (45.5%)			
≥20	18 (40.0%)	36 (54.5%)			
**Child-pugh**, ***n***			0.245	/	0.621
A	32 (71.1%)	44 (66.7%)			
B	13 (28.9%)	22 (33.3%)			
**HBV-DNA**, ***n***			0.072	/	0.789
Negative	39 (86.7%)	56 (84.8%)			
Positive	6 (13.3%)	10 (15.2%)			
**Tumor number**, ***n***			0.028	/	0.867
Single	30 (66.7%)	45 (68.2%)			
Multiple	15 (33.3%)	21 (31.8%)			
Tumor size^*^, ***n***			3.008	/	0.083
< 5 cm	40 (88.9%)	50 (75.8%)			
≥5 cm	5 (11.1%)	16 (24.2%)			
**Tumor size** ^*^ **, cm**					
(mean ± SD)	3.427 ± 1.044	3.556 ± 1.187	/	−0.585	0.56
**Liver cirrhosis**, ***n***			0.114	/	0.736
Yes	30 (66.7%)	46 (69.7%)			
No	15 (33.3%)	20 (30.3%)			
**Tumor capsule**, ***n***			0.001	/	0.975
Complete	24 (53.3%)	35 (53.0%)			
Incomplete	21 (46.7%)	31 (47.0%)			
**Tumor differentiation**, ***n***			0.005	/	0.944
Poor/moderate-poor	33 (73.3%)	48 (72.7%)			
Moderate	12 (26.7%)	18 (27.3%)			
**AJCC/UICC TNM stage**, ***n***			0.028	/	0.867
I	30 (66.7%)	45 (68.2%)			
II	15 (33.3%)	21 (31.8%)			

*n*, Number of patients; ^*^Tumor size was determined by the maximum diameter for single and cumulative diameters for multiple. All factors had no statistically significant value (*P* > 0.05). Cancer stage was classified according to the new AJCC/UICC TNM staging system.

**Table 3:** Comparison of the post-treatment features between repeated hepatectomy (RH) group B and the RFA group.

**Variable**	**RH group B (*n =* 45)**	**RFA group (*n =* 66)**	***U/t***	***P* valve**
Hospital time, days (mean ± SD)	6.5 ± 0.8	5.5 ± 1.1	5.208	< 0.001
Hospitalization cost, CNY (mean ± SD)	29,009 ± 3,806	29,944 ± 3,752	−1.282	0.202
**Postoperative markers**				
**ALT, IU/L, M (X**_25%_ **, X**_75%_ **)** ^*^				
The first day	303.3 (123.6, 361.5)	160.2 (110.5, 217.5)	846	< 0.001
The third day	148.8 (82.8, 181.2)	106.3 (56.0, 141.6)	990	0.003
The fifth day	47.4 (40.1, 61.9)	47.8 (31.8, 58.0)	1274	0.205
**AST, IU/L, M (X**_25%_ **, X**_75%_ **)** ^*^				
The first day	184.9 (135.4, 257.5)	157.8 (112.5, 227.1)	1282.5	0.224
The third day	63.6 (31.7, 120.5)	79.7 (39.0, 137.58)	1230.5	0.126
The fifth day	31.8 (23.2, 48.6)	30.9 (24.8, 45.6)	1464	0.9
**Total bilirubin (TB), g/L (mean** ±**SD)**				
The first day	36.5 ± 12.4	24.2 ± 8.7	6.151	< 0.001
The third day	20.4 ± 9.2	22.4 ± 9.4	−1.099	0.274
The fifth day	17.3 ± 8.2	20.3 ± 9.7	−1.663	0.099
**Direct bilirubin (DB)**, μ**mol/L (mean** ±**SD)**				
The first day	20.2 ± 7.4	11.5 ± 4.5	7.711	< 0.001
The third day	9.1 ± 3.0	10.6 ± 4.0	−2.109	0.037
The fifth day	7.4 ± 1.5	8.4 ± 2.7	−2.394	0.018
**Albumin (ALB), g/L (mean** ±**SD)**				
The first day	34.8 ± 3.0	36.6 ± 2.8	−3.041	0.003
The third day	36.5 ± 3.7	35.2 ± 3.6	1.8	0.075
The fifth day	38.6 ± 3.0	37.0 ± 3.5	2.504	0.014

*n*, Number of patients; Data were expressed as mean ± SD deviation; ^*^ALT/AST was presented as *M* (25%, 75%).

**Figure 2:**
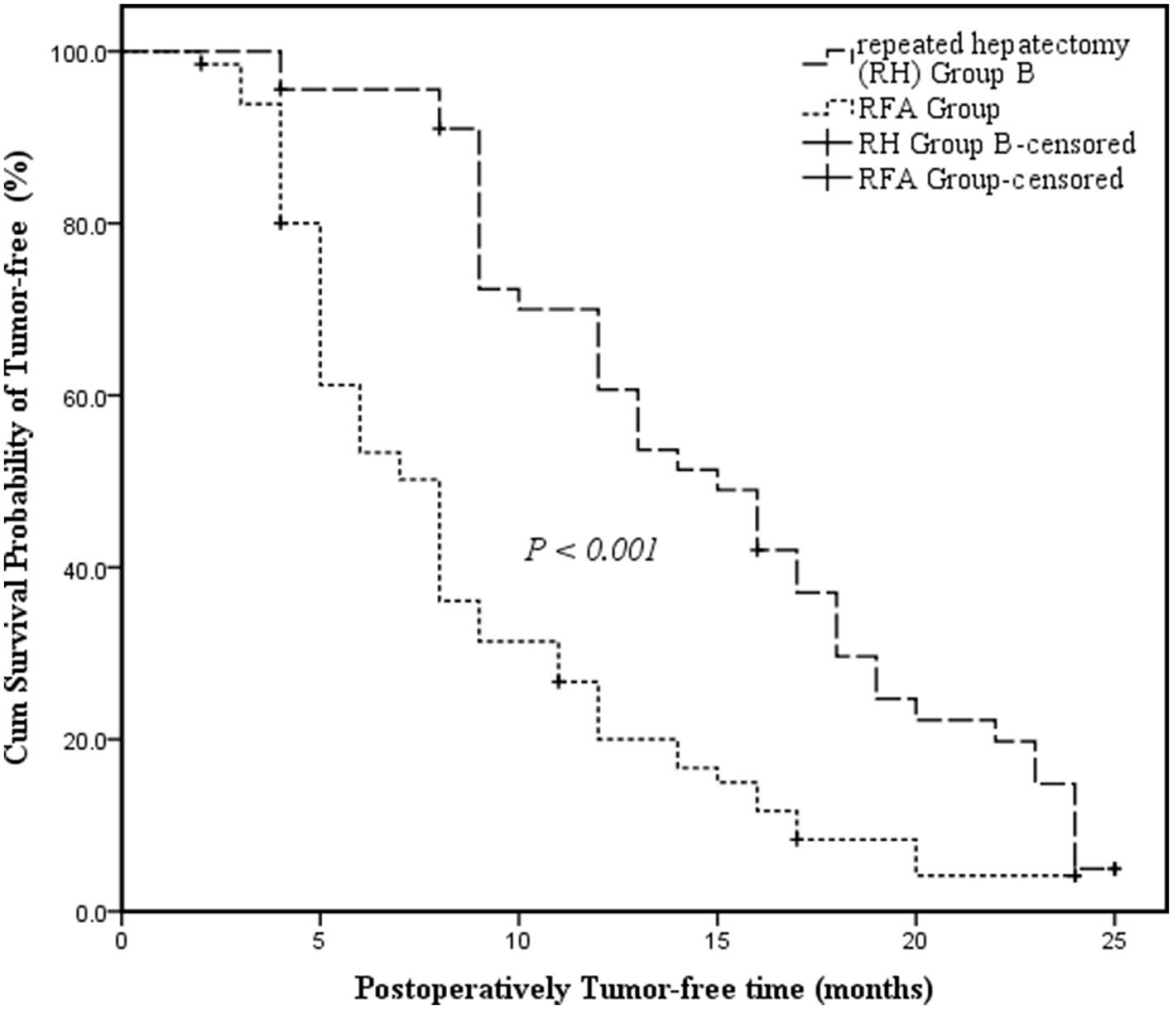
Kaplan-Meier survival curves assessing the cumulative tumor-free survival of Repeated Hepatectomy Group B and the RFA Group. The cumulative survival was compared between groups using the log-rank test. Kaplan-Meier medians: Repeated Hepatectomy (RH) Group B: 15 months (95% CI: 11.798–18.202); RFA Group: 8 months (95% CI: 6.593–9.407). The tumor-free survival of Repeated Hepatectomy Group B is significantly longer than that of the RFA Group (log-rank test: χ^2^ = 18.709, *P* < 0.001).

RH Group B had a longer median tumor-free survival time than the RFA Group and the survival curve revealed that tumor-free survival time differed significantly between RH Group B and the RFA Group (15 months, 95% CI: 11.798–18.202 vs. 8 months, 95% CI: 6.593–9.407; *P* < 0.001) (Figure 2).

### 3.3. Univariate and multivariate analyses of the 1-year postoperative tumor-free survival of recurrent HCC patients following RH

Univariate analysis revealed a significant positive correlation between age ≥50 y, Child-Pugh class A, HBV-DNA negative status, tumor size < 5 cm, a single tumor, a complete tumor capsule and regular resection, and 1-year postoperative tumor-free survival among RH Group A patients with recurrent HCC (*P* = 0.032, *P* = 0.026, *P* = 0.004, *P* = 0.003, *P* = 0.006, *P* < 0.001 and *P* = 0.013, respectively) (Table 4). Multivariate analysis indicated that age, Child-Pugh score, and HBV-DNA were independent risk factors of survival (HR = 0.170, 95% CI: 0.075–0.385, *P* < 0.001; HR = 5.842, 95% CI: 2.406–14.187, *P* < 0.001; HR = 5.558, 95% CI: 2.464–12.539, *P* < 0.001, respectively) (Table 5).

**Table 4:** Univariate analysis of the 1-year postoperative tumor-free survival rate of the repeated hepatectomy (RH) for recurrent HCC (RH group A, *n* = 84).

**Variable**	**Assignment**	***n***	Tumor-free survival rate (1st Year) *n*/%^*^	**χ^2^**	***P* valve**
**Age, y**, ***n***				4.617	0.032
< 50	0	32 (38.1%)	10/31.3%		
≥50	1	52 (61.9%)	29/55.8%		
**Gender**, ***n***				1.33	0.249
Female	0	18 (21.4%)	6/33.3%		
Male	1	66 (78.6%)	33/50.0%		
**AFP, ng/ml**, ***n***				1.534	0.215
< 20	0	44 (52.4%)	23/52.3%		
≥20	1	40 (47.6%)	16/40.0%		
**Child-Pugh**, ***n***				4.976	0.026
A	0	56 (66.7%)	31/55.4%		
B	1	28 (33.3%)	8/28.6%		
**HBV-DNA**, ***n***				8.23	0.004
Negative	0	62 (73.8%)	33/53.2%		
Positive	1	22 (26.2%)	6/27.3%		
**Tumor number**, ***n***				7.575	0.006
Single	0	39 (46.4%)	24/61.5%		
Multiple	1	45 (53.6%)	15/33.3%		
**Tumor size**, ***n***				8.552	0.003
< 5 cm	0	51 (60.7%)	30/58.8%		
≥5 cm	1	33 (39.3%)	9/27.3%		
**Liver cirrhosis**, ***n***				0.077	0.781
No	0	15 (17.9%)	6/40.0%		
Yes	1	69 (82.1%)	33/47.8%		
**Tumor capsule**, ***n***				12.331	< 0.001
Complete	0	35 (41.7%)	24/68.6%		
Incomplete	1	49 (58.3%)	15/30.6%		
**Tumor differentiation**, ***n***				0.37	0.831
Well/well-moderate	0	3 (3.6%)	2/66.7%		
Moderate	1	23 (27.4%)	9/39.1%		
Poor/moderate-poor	2	58 (69.0%)	28/48.3%		
**Surgical approach, n**				6.17	0.013
Regular liver resection	0	21 (25.0%)	15/71.4%		
Irregular liver resection	1	63 (75.0%)	24/38.1%		

*n*, Number of patients in repeated hepatectomy (RH) Group A; ^*^Data was expressed as number of patients (% of Postoperative Tumor-free survival rate in the 1^st^ year).

**Table 5:** Multivariate analysis of the 1-year postoperative tumor-free survival rate of repeated hepatectomy (RH) for recurrent HCC.

**Variable**	**Standard regression coefficient (B)**	**Standard error (SE)**	**Wald (*W*)**	***P* valve**	**Hazard ratio [Exp (B)]**	**95% CI for Exp (B)**	
						**Lower**	**Upper**
Age	−1.772	0.418	17.994	< 0.001	0.170	0.075	0.385
Child-Pugh	1.765	0.453	15.207	< 0.001	5.842	2.406	14.187
HBV-DNA	1.715	0.415	17.078	< 0.001	5.558	2.464	12.539
AFP	−0.443	0.350	1.601	0.206	0.642	0.323	1.275
Tumor number	0.371	0.463	0.643	0.423	1.450	0.585	3.591
Tumor size	0.479	0.443	1.172	0.279	1.615	0.678	3.848
Tumor capsule	1.031	0.562	3.366	0.067	2.804	0.932	8.435
Surgical approach	0.615	0.654	0.884	0.347	1.849	0.513	6.662

The variables contained the eligible factors including the elements of clinical significance.

## 4. Discussion

Cirrhosis underlies HCC in approximately 90% of HCC cases. While the most common causes of cirrhosis leading to HCC include alcohol-related liver disease (ALD), non-alcoholic fatty liver disease (NAFLD), or non-alcoholic steatohepatitis (NASH), and viral hepatitis (hepatitis B [HBV] and hepatitis C [HCV]) (1), the most common cause of HCC in the absence of cirrhosis is HBV infection. More recently, there has also been an increase in non-cirrhotic NAFLD-related HCC (3). A reduction in viral hepatitis-related HCC cases in certain parts of the world has been offset by an increase in alcohol and non-NAFLD-related cases. Advances in medical technology and intensive care have improved the therapeutic options for HCC in recent years (10). Treatment options are broadly categorized as curative (i.e., liver transplantation, resection, or ablation/segmental transarterial radioembolization [TARE]) and non-curative (transarterial chemoembolization [TACE] or systemic therapies) (1).

HCC staging is dependent on performance status (PS), degree of liver dysfunction, and traditional TNM staging. To better characterize prognosis and available treatment options, the American Association for the Study of Liver Diseases (AASLD) and the European Association for the Study of the Liver (EASL) recommended updates to the Barcelona Clinic Liver Cancer (BCLC) classification in 2022 (11). Liver function is now characterized as compensated or decompensated with or without jaundice, ascites, and/or hepatic encephalopathy regardless of Model for End-Stage Liver Disease (MELD) or Child-Pugh (CP) classification. It is recommended that individuals with cirrhosis, a significant degree of liver dysfunction, or clinically significant portal hypertension (CSPH), without medical or psychosocial contraindications, pursue liver transplantation. The Milan criteria (MC) has been successfully used as the selection tool for identifying appropriate orthotopic liver transplantation (OLT) candidates based on tumor size and number including one lesion >2 cm and < 5 cm or ≤ 3 lesions of >1 cm and < 3 cm with no vascular invasion or additional extrahepatic spread. While OLT is the most definitive treatment for HCC, organ shortage limits this option.

Resection is generally recommended for early-stage HCC patients without stage VI, and outcomes which are especially relevant in individuals with appropriate candidates for resection are comparable to those of transplant recipients (1). While not performed as frequently as the conventional treatment option, surgical resection of larger tumors, including low-grade VI or portal vein tumor thrombus (PVTT), has also been performed with successful outcomes (12). However, the 5-year overall survival (OS) rate of HCC remains at only 12% (13). This is attributed to the high risk of recurrence after resection, with an annual recurrence rate of 10% and a 5-year recurrence of 70–80% (14). Fortunately, there are several treatment options for recurrent HCC patients, including resection (i.e., RH), RFA, and salvage transplantation (SLT) (15). SLT is particularly effective at treating recurrent HCC (16–18), however, due to the lack of donor resources, the clinical use of SLT is limited (19, 20). As a result, no satisfactory treatments are being used to select appropriate recurrent HCC candidates. Recent studies indicate that RH is the preferred treatment modality for recurrent HCC. In 1986, Nagasue et al. were the first to report the use of RH among nine recurrent HCC patients with no surgical death (21). Another study found that the hospitalization mortality rate was only 1% in 94 patients with RH, including eight who underwent a third hepatectomy and two who underwent a fourth hepatectomy (22). While RH can be associated with severe technological challenges, including intra-abdominal adhesions caused by the initial operation, studies indicate that RH is a feasible method for recurrent HCC patients undergoing IH (23–25). Thus, RH could be a preferred treatment option for patients with recurrent HCC.

In recent years, laparoscopic repeat hepatectomy (LRH) has been used in the clinical setting. As treatment for recurrent HCC, LRH has obvious advantages, including earlier recovery of liver function, less blood loss, and shorter hospital stays (26–28). However, the success of LRH can be challenged by the presence of densely formed adhesions from previous operations (26). Fortunately, this advanced surgical technique is reserved for appropriate candidates by our surgical teams.

The current study has also provided vital links between the IH Group and RH Group A. While the clinical pathology of patients in the RH Group A was inferior to those in the IH Group, including tumor number, liver cirrhosis, tumor differentiation, surgical approach, and TNM stage (all *P* < 0.001), as well as the operation time (*P* < 0.001), there was no significant difference in the risk of intraoperative bleeding (*P* = 0.204), indicating that RH for recurrent HCC is safe and effective. In addition, the tumor-free survival of RH Group A was visibly lower than that of the IH Group (12 months, 95% CI: 9.474–14.526 vs. 22 months, 95% CI: 14.651–29.349; *P* < 0.001) (Figure 1). These findings indicated that patients with recurrent tumors undergoing RH may be more susceptible to relapse than those with initial tumors undergoing IH.

Thermal ablation, including microwave ablation (MWA), RFA, cryotherapy, laser interstitial thermal therapy (LITT), and alcohol or electroporation, is an appropriate therapeutic approach for tumors that are 3–4 cm in diameter or less. MWA (for tumors up to 4 cm) and RFA (for tumors up to 3 cm) are most used in clinical practice and have the advantage of only requiring minor invasion. Both techniques are comparable to resection for smaller tumors (< 2 cm) and should be the first-line approach according to AASLD, EASL, and the Asian-Pacific Association for the Study of the Liver (APASL) guidelines (29). Ablation should be performed to achieve a 10-mm margin around the tumor to maximize the removal of any microsatellite lesions that predominantly occur within 10 mm of HCC lesions. RFA is more suitable for ≤ 3 recurrent HCC lesions that are ≤ 3 cm in diameter (22, 30, 31). According to ablative criteria and indications, the application for RFA to ensure success is extremely severe.

To evaluate the efficacy of RH treatment for recurrent HCC, several indicators were compared between the RH Group B and RFA Group. While hospital time differed significantly between the two groups (RH Group B vs. RFA Group: 6.5 ± 0.8 d vs. 5.5 ± 1.1 d, *P* < 0.001), hospitalization cost was comparable (*P* = 0.202). These findings indicated that, compared to RH, RFA has the advantage of being minimally invasive, leading to enhanced recovery after surgery (ERAS), and the disadvantage of requiring an expensive apparatus that elevates costs. However, a recent meta-analysis (32) showed that local ablative techniques resulted in a similar OS but improved recurrence-free survival (RFS) and local recurrence rates beyond surgical resection (HR = 0.75; 95%CI: 0.65–0.96). Meanwhile, resection led to longer OS and RFS than WMA and RFA + TACE. Therefore, RH might be an ideal option for prolonging the OS of individuals with recurrent HCC (33). Fortunately, our research found that the cumulate survival probability of tumor-free and postoperative tumor-free survival time were higher among RH Group B patients than those in the RFA Group (15 months, 95% CI: 11.798–18.202 vs. 8 months, 95% CI: 6.593–9.407; *P* < 0.001) (Figure 2). Meanwhile, the measurements of all characteristics were similar between the two groups, suggesting that the pre-treatment patients had equal status (all *P* > 0.05). The RH Group B outcomes indicated that the therapeutic effect of RH leads to better tumor control than RFA treatment. This may be because RFA treatment is performed in the absence of an adequate margin of < 10 mm surrounding the tumor. This could fail to completely ablate tumor boundaries due to factors such as tumor location, risk of tumor seeding, and inability to obtain tumor samples, which were not considered. Thus, the clinical application for RFA treatment is limited to a certain extent.

The postoperative biomarkers of liver function indicated that while ALT levels were statistically different between RH Group B and the RFA group on day 1 and 3 (*P* < 0.001, *P* = 0.003), there was no obvious difference in ALT levels on day 5 (*P* = 0.205) or in AST levels on day 1, 3, and 5 (all *P* > 0.05). These results suggested that the postoperative influence of the two treatments on ALT and AST levels was not obvious 5 days after surgery. The findings were similar for TB. Furthermore, DB levels on day 1, 3, and 5, and ALB levels on day 1 and 5 were both statistically significant (all *P* < 0.05). However, DB levels on day 1 and 3 and ALB levels on day 1 were worse in the RH Group B than the RFA group but were better by day 5.

It is also important to consider the effect of postoperative exogenous supplementation on albumin levels. Fortunately, the current study showed that the biochemical indices of liver function were not vastly different 5 days after RH and RFA. Thus, it is likely that RH did not further impair liver function compared to RFA, which had the advantage of being minimally invasive. We believe that RH may be preferable to RFA for recurrent HCC patients with severe indications.

Chan et al. (34) found that time to recurrence (TTR) and recurrence in more than one organ were the influencing factors for OS after hepatectomy for primary HCC. Univariate and multivariate analyses indicated that recurrence in more than one organ remained an independent unfavorable prognostic factor for OS. The risk of recurrence after initial resection for primary HCC depends on tumor differentiation, the presence of microvascular invasion, and tumor size or burden (14). The current study used univariate and multivariate analyses to explore the risk factors affecting the 1-year postoperative tumor-free survival among patients with recurrent HCC undergoing RH. Age ≥50 y, Child-Pugh Class A, and negative HBV-DNA status were identified as independent risk factors that beneficially impacted the 1-year postoperative tumor-free survival rate (*P* < 0.001, *P* < 0.001, *P* < 0.001, respectively). These findings suggest that a better target organ of the liver could play a more important role than pathology in predicting the recurrence of HCC patients undergoing RH. Thus, more attention should be placed on improving hepatic function and treating HBV, particularly among recurrent HCC patients undergoing RH.

Since the concept of downstaging (DS) to the MC was first published by the University of California, San Francisco (UCSF) group in 2008, additional data has supported DS as a viable approach to access OLT with excellent post-LT results (35). In general, DS is used to gain insight into the biological activity of tumors exceeding the MC based on their response to locoregional therapies (LRTs). A pivotal prospective study (36) established the role of stereotactic body radiation therapy (SBRT) along with embolization techniques in downstaging patients with HCC and PVTT as a bridge to successful living donor transplantation. There have been tremendous advancements in transplant and resection eligibility following acceptance of the “DS” concept. First, multiple embolization techniques, including transarterial bland embolization (TAE), TACE, drug-eluting bead TACE (DEB-TACE), and TARE, should be considered as additional valuable care on DS. For example, TACE is the current standard of care for intermediate-stage HCC (BCLC B) (37) and can be used to promote survival or aid DS as a bridge to transplant or resection. Second, since sorafenib was approved for advanced HCC in 2007, no other systemic agents have been approved. While several single first-(lenvatinib) and second-line (regorefenib, cabozantinib, ramucirumab, and pembrolizumab) agents have been approved, combination therapy has demonstrated the most promising results for advanced HCC, including the IMBRAVE 150 in 2019 and the HIMALAYA trial in 2022. Tyrosine kinase inhibitors (TKIs) are shown to decrease tumor resistance to immunotherapy and are being studied in combination with immunotherapy agents. A pharmacological class known as immune checkpoint inhibitors (ICIs) has also been developed as a potential treatment option for various malignancies, including HCC. ICIs have significant clinical advantages as either mono or combination therapies. TKIs decrease tumor resistance to immunotherapy and are being studied in combination with immunotherapy agents. While the use TKIs as monotherapy may decline, they remain an important therapeutic option for patients who are ineligible for immunotherapy and may prove to be an efficacious adjunct to immunotherapy. There may also be a role for combination immunotherapy, TKIs, and anti-VEGF therapies as a complement to ablative and embolization techniques. Importantly, an increase in the number of agents used in combination must be balanced with the risk of increased adverse events and reduced tolerability even in patients with well compensated cirrhosis. The combination of LRTs and systemic therapies (either in the neoadjuvant or the adjuvant setting) or combinational systemic therapies, including doublets of ICIs, ICIs + TKIs, and ICIs + bevacizumab, could be used to downstage HCC as a “bridge” to OLT or SLT (improve the staging of HCC beyond MC to within MC) and IH or RH.

It is clear that RFA is mostly suitable for patients with multiple lesions (≤ 3) and tumors ≤ 3 cm in diameter (30, 31). While SLT produces the best survival results, this treatment is limited by the lack of liver graft availability (19, 20). Moreover, RH shows better survival outcomes than TACE for recurrent HCC (38, 39). Combination systemic therapies, including immunotherapy and TKIs, are being increasingly used for advanced liver cancer patients who are ineligible for hepatectomy. However, while the potential role of LRTs combined with systemic therapy, which is limited to BCLC A and B, has been strengthened, there is still no satisfactory option for the total effective rate of the remedies which benefits HCC patients, including initials and recurrences. Thus, RH is widely accepted as the preferred treatment for recurrent HCC patients with compensated liver function reserves (15, 40).

The current study demonstrated that RH could be performed safely and was able to improve the tumor-free survival of patients with recurrent HCC. Moreover, in recurrent HCC patients undergoing RH, a well-functioning liver may play a more important role than lesion pathology. The superior outcomes of RH for recurrent HCC may inform treatment options for this patient population.

It is important to note that the longest tumor-free survival time of recurrent HCC patients receiving RH was only 25 months in this study (Figures 1, 2). This shows both the value of RH and its limitations in preventing early intrahepatic recurrence. Thus, it will be important to further explore comprehensive treatment options for recurrent HCC. The more ideal trial design will include a centralized multidisciplinary team (MDT) with successful DS defined as complete response (CR) and a longer period of observation before randomization [6 months is required by the United Network for Organ Sharing (UNOS) post-DS to MC] and the inclusion of explant pathology in the analysis (41). Fortunately, immunotherapy has transformed the treatment of every stage of HCC and has the potential to complement most other therapeutic approaches. Furthermore, advances in curative and non-curative treatment options and the evaluation of combination treatments, including the use of LRTs and systemic therapies to “down-stage” individuals to reach LT or RH eligibility, will inform how to best manage HCC recurrence. These therapeutic innovations are accompanied by the challenge of rigorously comparing various treatment strategies. The current study also has some pitfalls, such as its small sample size, the retrospective nature of the design, and the use of a single center. A randomized prospective study is needed to further validate the research findings.

## Data availability statement

The original contributions presented in the study are included in the article/supplementary material, further inquiries can be directed to the corresponding authors.

## Ethics statement

The study was reviewed and approved by the Medical Ethics Committee of Huzhou Central Hospital and the application for informed patient consent was exempted.

## Author contributions

QY participated in the design. XS and ZP get the data collection, analyses, and manuscript preparation. FC performed statistical analysis, maintained the primary database, and also was involved in critical appraisal of the study data as well as editing of the manuscript. All authors contributed to the article and approved the submitted version.
